# Quality assessment of the guidelines for the management of malignant pleural effusions and ascites

**DOI:** 10.1186/s12957-020-02097-y

**Published:** 2020-12-11

**Authors:** Jia-Xi Li, Yan-Mei Shi, Li-Ya An, Jin-Xu Yang, Yu-Xing Qi, Ting Yang, Yun-Yun Cen, Yue-Ying Lin, Da-Li Sun

**Affiliations:** 1 Department of Gastrointestinal Surgery, Second Affiliated Hospital of Kunming Medical University/Second Faculty of Clinical Medicine, Kunming Medical University, Kunming, 650101 China; 2 Department of Gastroenterology, Second Affiliated Hospital of Kunming Medical University/Second Faculty of Clinical Medicine, Kunming Medical University, Kunming, 650101 China

**Keywords:** Malignant pleural effusion, Malignant ascites, Guideline appraisal

## Abstract

**Objectives:**

To fully assess the quality of the guidelines for the management of malignant pleural effusions (MPE) and ascites and reveal the heterogeneity of recommendations and possible reasons among guidelines.

**Methods:**

A systematic search was performed in the database to obtain guidelines for the management of MPE and ascites. The AGREE IIGtool was used to assess the quality of these guidelines. The Measurement Scale of Rate of Agreement (MSRA) was introduced to assess the scientific agreement of formulated recommendations for the management of MPE and ascites among guidelines, and evidence supporting these recommendations was extracted and analyzed.

**Results:**

Nine guidelines were identified. Only 4 guidelines scored more than 60% and are worth recommending. Recommendations were also heterogeneous among guidelines for the management of MPE, and the main reasons were the different emphases of the recommendations for the treatment of MPE, the contradictions in recommendations, and the unreasonably cited evidence for MPE.

**Conclusions:**

The quality of the management guidelines for patients with MPE and malignant ascites was highly variable. Specific improvement of the factors leading to the heterogeneity of recommendations will be a reasonable and effective way for developers to upgrade the recommendations in the guidelines for MPE.

## Background

Malignant pleural effusion and malignant ascites are common complications of malignant tumors, often indicating that malignant tumors have reached an advanced stage and thus leading to a shorter survival and lower quality of life. Malignant pleural effusion (MPE) refers to pleural effusion caused by the metastasis of malignant tumors from the pleura or other location to the pleura. The median survival is approximately 3 to 12 months. Most commonly, cases of MPE originate from lung cancer (37.5%), followed by breast cancer (16.8%) and lymphoma (11.5%) [1]. Malignant ascites refers to peritoneal effusion caused by primary peritoneal or other malignant tumors that metastasize to the peritoneum. Malignant ascites is most common in ovarian cancer (37%), followed by liver-biliary and pancreatic tumors (21%) and gastric cancer (18%). Excluding ovarian cancer, the median survival is approximately 5.7 months [1]. Patients with malignant pleural effusion and malignant ascites have a poor prognosis, low quality of life, and high mortality, and the management of these conditions is a challenging but important issue. At present, the management of malignant pleural effusion and malignant ascites has received extensive attention from various academic institutions and experts, and relevant guidelines have been formulated to regulate clinical work [1–9]. However, the recommended contents of these guidelines vary greatly, and the quality is uneven, making these guidelines unsuitable for use.

The Appraisal of Guidelines for Research and Evaluation II (AGREE II) is a reliable and useful tool for assessment of guidelines. This tool has been validated in guideline evaluation studies in critical care medicine, pain medicine, urinary system diseases, nutrition support, etc. [10–14]. Currently, there is no guideline appraisal for the management of malignant pleural effusion and ascites available.

Therefore, to provide some useful references for guideline makers, we decided to use the AGREE ertool to conduct a comprehensive evaluation of nearly 10 years of malignant pleural effusions and malignant ascites guidelines, analyze the key recommendations of the guidelines, identify heterogeneities, and find the reasons for these differences.

## Methods

This study was carried out in accordance with the Preferred Reporting Items for Systematic Reviews and Meta-Analyses (PRISMA). AGREE II instrument was introduced to conduct a comprehensive evaluation of the methodological quality of the guidelines for the management of malignant pleural effusions and malignant ascites and to evaluate the rigor and transparency of the management of malignant pleural effusions and malignant ascites.

### Search strategy

A systematic search for guidelines on malignant pleural effusion and malignant ascites was performed from October to November 2019 without time and language limitations in our study. The databases we searched included PubMed (https://www.ncbi.nlm.nih.gov/pubmed/), Web of Science (http://apps.webofknowledge.com/), and CNKI (https://www.cnki.net). Furthermore, some relevant websites were also searched to prevent omitting guidelines, such as Baidu (www.baidu.com), NCCN (https://www.nccn.org/professionals/physian_gls/f_guldelines.asp), ATS journal (https://www.atsjournals.org), ERS journal (https://erj.ersjournals.com), and BTS (https://brit-thoracic.org.uk). The search keywords were “malignant pleural effusion/malignant ascites”, and the limitations were “Title/Abstract” and “guideline/statement/consensus/recommendation”.

### Inclusion criteria and exclusion criteria

We applied a number of inclusion criteria to ensure that we screened the appropriate guidelines: (1) must be a guideline, recommendation, statement, or consensus; (2) must have a clear and detailed recommendation for the management of malignant pleural effusion and malignant ascites in sections or full text; (3) issued within the last decade; and (4) was the latest version. Moreover, the collected guidelines were excluded according to the following criteria: (1) duplicate publications; (2) old version of the guidelines; and (3) an interpretation or assessment of guidelines rather than guidelines itself.

A total of two reviewers independently evaluated the search results (L.J.X., Y.J.X.) to determine whether to include or exclude each reference, in case of disagreement, a discussion with a third expert reviewer was conducted.

### Quality appraisal of the guidelines

The tool we used to objectively assess the quality of the guidelines is the AGREE II tool (2017 version, http://www.agreetrust.org/), which covers 23 items in six domains. The score of each domain can be used to judge the merits and recommendation level of the guidelines [15].

*Domain 1. Scope and purpose*. There are 3 items, including the overall subject, target population, and health issues involved.

*Domain 2. Stakeholder involvement*. This domain consists of 3 items, which list the participants, the views of the patients, and the target users.

*Domain 3. Rigor of development*. A total of 8 items comprise this domain, including systematic search, criteria for evidence selection, merits of evidence, formulation methods of the guidelines, consideration of health benefits and risks, clear connection between recommendations and supporting evidence, external expert review before publication and update to the guidelines.

*Domain 4. Clarity and presentation*. There are 3 items, including clear recommendations, various options for the same question, and easy-to-distinguish main recommendations.

*Domain 5. Applicability*. Four items are included: promotions and obstacles in the process of application, relevant supporting documents, potential resource costs, and monitoring and auditing standards.

*Domain 6. Editorial independence*. Two items comprise this domain: sponsors and funding and conflicts of interest.

Four of our experienced and trained members (L.J.X., Y.J.X., A.L.Y, S.Y. M) rated each of the 23 items and six domains on a scale of 1 to 7, with “1” indicating total noncompliance and “7” indicating total compliance. The higher the score is, the better the quality. Any difference in score of more than 2 to 3 points for an item was discussed to determine the final score, and then one of our members summarized and calculated the scores of each domain:

(obtained score − minimum possible score)/(maximum possible score − minimum possible score) × 100%.

Finally, the weighted mean of the six domains was taken to obtain the final score of the guidelines. We conventionally defined a score greater than 60% as a recommendation for the guidelines, a score of less than 30% as no recommendation, and a score between 30 and 60% as a recommendation, but the guideline needs to be revised.

### Statistical analysis

This is a descriptive study, and SPSS version 17.0 (SPSS, Chicago, USA) was used for two-way ANOVA to calculate the ICC (i.e., measurement and evaluation index difference between observers). The ICC is normally between 0 and ~ 1, and the higher the value is, the better the reliability. Good consistency among 2~4 people can be considered when ICC > 0.75, and *P* < 0.05 indicates a significant difference.

## Results

**Fig. 1:**
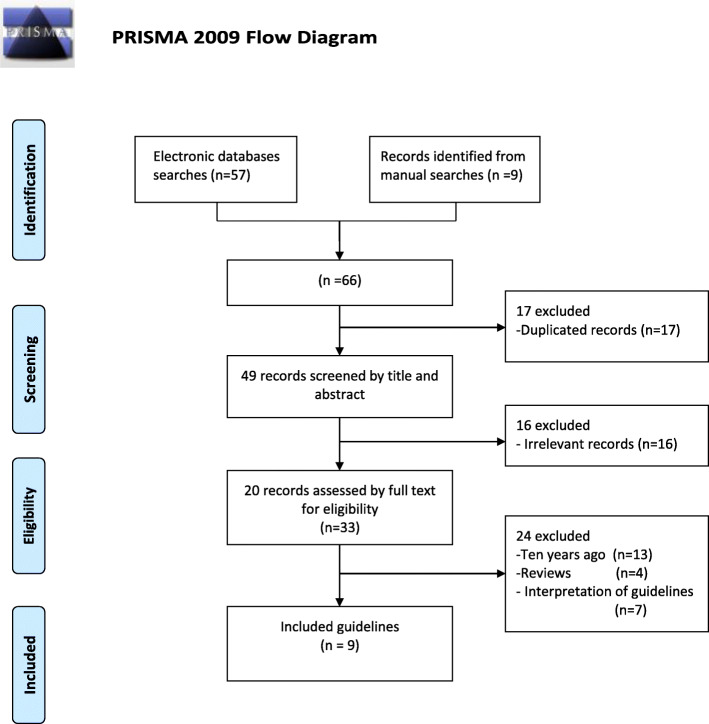
PRISMA 2009 flow diagram

According to the above search strategy, a total of 66 articles were obtained from the database and manual search, among which 17 were duplicate articles. After removing the duplicate articles, only 49 articles were left, and then these articles were screened one by one according to the inclusion and exclusion criteria. A total of 9 articles were finally identified (see Fig. 1 and Table 1).

**Table 1:** Characteristics of included guidelines

Guideline ID	Organization	Country/area	Version	Topic	Grading system	Method
ATS, 2018 [5]	ATS/STS/STR	America	Original	Management of malignant pleural effusions	GRADE	EB
ERS, 2018 [2]	ERS/EACTS	Europe	Original	Management of malignant pleural effusions	Unclear	EB
BTS, 2010 [3]	BTS	Britain	Original	Management of malignant pleural effusions	Unclear	EB
CGDTMPE, 2014 [4]	CGDTMPE	China	Original	Malignant pleural effusions	Unclear	CB
CSCO, 2018 [1]	CSCO	China	Original	Administration of rmhTNF for malignant ascites	Unclear	EB
CTS, 2009 [6]	CTS	China	Original	Respiratory diseases	Unclear	CB
JSPM1, 2016 [7]	JSPM	Japan	Original	Parenteral fluid management for terminal cancer patients	GRADE	CB
JSPM2, 2016 [8]	JSPM	Japan	Original	Treatment recommendations for respiratory symptoms in cancer patients	GRADE	EB
JSPM, 2019 [9]	JSPM	Japan	Original	Gastrointestinal symptoms in cancer patients	GRADE	EB

*ATS* American Thoracic Society, *STS* Society of Thoracic Surgeons, *STR* Society of Thoracic Radiology, *ERS* European Respiratory Society, *EACTS* European Association for Cardio-Thoracic Surgery, *BTS* British Thoracic Society, *JSPM* Japanese Society for Palliative Medicine, *CSCO* Chinese Society of Clinical Oncology, *CGDTMPE* Chinese Expert Consensus Group on Diagnosis and Treatment of Malignant Pleural Effusion, *CTS* Chinese Thoracic Society, *CB* Consensus based, *EB* Evidence based

### Characteristics of the included guidelines

We have summarized the characteristics of these 9 guidelines (Table 1), which cover a span of 10 years from 2009 to 2018; three are from Japan [7–9], one from the USA [5], one from Europe [2], another from the UK [3], and three are expert consensuses from China [1, 4, 6]. Among them, 8 guidelines [1–8] mentioned a specific management recommendation for malignant pleural effusion, while 3 guidelines [1, 7, 9] mentioned a clear management recommendation for malignant ascites. A total of 4 guidelines [5, 7–9] used GRADE as an evidence grading system, 5 guideline did not clearly describe the evidence grading system [1–4, 6], and 6 guidelines [1–3, 5, 8, 9] were evidence-based guidelines.

### Quality assessment of the guidelines

We assessed these nine guidelines according to the latest version of the AGREE itool (see Table 2). Regarding the median score of all guidelines across various domains, the scope and purpose scored 88.9% (ranging from 50.0 to 94.4%), stakeholder involvement scored 47.2% (ranging from 30.6 to 66.7%), rigor of development scored 54.7% (ranging from 26.0 to 85.4%), clarity and presentation scored 91.7% (ranging from 79.2 to 94.4%), the applicability scored 36.5% (ranging from 24.0 to 62.5%), and editorial independence scored 47.9% (ranging from 0.0 to 52.1%). Four guidelines that scored over 60% [2, 5, 8, 9] are worth recommending. The scores of the other 5 guidelines were between 30 and 60% [3–7]. The ICC values of all 9 guidelines were greater than 0.8.

**Table 2:** AGREE II domain score and ICC score of included guidelines

Guideline ID	Scope and purpose	Stakeholder involvement	Rigor of development	Clarity and presentation	Applicability	Editorial independence	Overall assessment	
ATS, 2018 [5]	91.7%	48.6%	54.7%	94.4%	62.5%	45.8%	64.1%	R
ERS, 2018 [2]	84.7%	62.5%	75.0%	79.2%	41.7%	50.0%	63.7%	R
BTS, 2010 [3]	50.0%	36.1%	33.3%	93.1%	41.7%	47.9%	47.1%	RM
CGDTMPE, 2014 [4]	54.2%	30.6%	26.0%	88.9%	25.0%	0.0%	34.5%	RM
CSCO, 2018 [1]	69.4%	47.2%	41.1%	91.7%	42.7%	0.0%	47.0%	RM
CTS, 2009 [6]	66.7%	58.3%	29.7%	93.1%	36.5%	0.0%	43.8%	RM
JSPM1, 2016 [7]	88.9%	33.3%	69.8%	88.9%	24.0%	52.1%	56.3%	RM
JSPM2, 2016 [8]	94.4%	66.7%	71.4%	88.9%	24.0%	47.9%	61.1%	R
JSPM, 2019 [9]	90.3%	41.7%	85.4%	94.4%	25.0%	47.9%	61.9%	R
ICC (mean ± SD)	0.94 ± 0.08	0.99 ± 0.008	0.98 ± 0.01	0.88 ± 0.08	0.99 ± 0.01	0.99 ± 0.002	—	—
Median score (range)	88.9% (50.0~94.4%)	47.2% (30.6~66.7%)	54.7% (26.0~85.4%)	91.7% (79.2~94.4%)	36.5% (24.0~62.5%)	47.9% (0~52.1%)	—	—

*R* Recommended, *RM* Recommended with modifications, *NR* Not recommended

### Heterogeneity of the recommendations and evidence of the included guidelines

To compare the differences in recommendations among the included guidelines for the management of malignant pleural effusions, we selected the ATS/STS/STR 2018 guidelines [5] (total score 64.4%) as the reference guidelines, which had the highest quality (see Table 2) among the guidelines about malignant pleural effusion according to our assessment. The various recommendations of the other seven guidelines were compared to those of the ATS/STS/STR 2018 guidelines (see Tables 3 and 4).

**Table 3:**
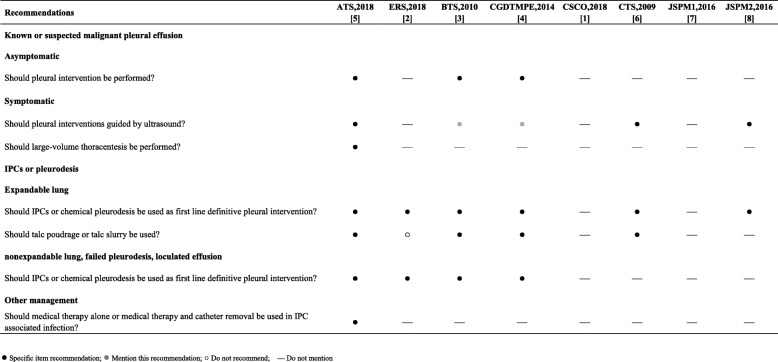
Recommendations for management of malignant pleural effusion

**Table 4:** Scientific agreement of formulated recommendations for management of malignant pleural effusion

Recommendations	ATS, 2018 [5]	ERS, 2018 [2]	BTS, 2010 [3]	CGDTMPE, 2014 [4]	CSCO, 2018 [1]	CTS, 2009 [6]	JSPM1, 2016 [7]	JSPM2, 2016 [8]
**Known or suspected malignant pleural effusion**								
**Asymptomatic**								
Should pleural intervention be performed?	—	—	80~100%	80~100%	—	—	—	—
**Symptomatic**								
Should pleural interventions guided by ultrasound?	—	—	80~100%	80~100%	—	80~100%	—	80~100%
Should large-volume thoracentesis be performed?	—	—	—	—	—	—	—	—
**IPCs or pleurodesis**								
**Expandable lung**								
Should IPCs or chemical pleurodesis be used as first-line definitive pleural intervention?	—	80~100%	40~60%	40~60%	—	40~60%	—	40~60%
Should talc poudrage or talc slurry be used?	—	0~20%	80~100%	80~100%	—	40~60%	—	—
**Nonexpandable lung, failed pleurodesis, loculated effusion**								
Should IPCs or chemical pleurodesis be used as first-line definitive pleural intervention?	—	80~100%	80~100%	80~100%	—	—	—	—
**Other management**								
Should medical therapy alone or medical therapy and catheter removal be used in IPC-associated infection?	—	—	—	—	—	—	—	—

Measurement Scale of Rate of Agreement: *0~20%* radically different, *20~40%* numerous major scientific disagreements present, *40~60%* few major scientific disagreements present, *60~80%* only minor scientific disagreements present, and *80~100%* absolute scientific agreement. In blank fields, no information is available

However, in the evaluation of malignant ascites, we only searched three guidelines about diagnosis and treatment: one set of guidelines [1] is aimed at rmhTNF treatment, one set of guidelines [9] is for the management of malignant ascites, and one set of guidelines [7] focuses on fluid management for patients with malignant ascites. We did not make a comparison among the recommendations of the three guidelines due to the different contents they emphasized.

## Discussion

### Main findings

Making guidelines for malignant pleural effusion and malignant ascites is a complex process. In this study, significant heterogeneity was found in guideline quality, recommendations, and level of evidence among the included guidelines, and even within the same guidelines. The main reasons for the heterogeneity among these guidelines are the different emphases of the guidelines, contradicting recommendations and unreasonably cited evidence.

### Quality assessment for the guidelines according to AGREE II

The findings of quality assessment for the guidelines in this study are similar to the results in previous studies [10, 12, 14], which are mainly related to the different methodologies adopted by different guideline development teams.

Domain 1, scope and purpose, is involved in each guideline because it covers the most basic feature of the guidelines, including the purpose for which they were developed, the major health issues involved, the main recommendations, and the target population.

In domain 2, stakeholder involvement, all the guidelines failed to take into account the views of the target population. However, in today's patient-oriented world, guidelines should be more considerate of patients and consider their opinions and ideas, which can not only improve the doctor-patient relationship but also improve the standard of the guidelines. Therefore, it is necessary to take measures to include opinions from the target population, including through questionnaires, literature searches, and telephone calls, and then incorporate the results in the guidelines.

Rigor of development is a domain with great differences among guidelines, and the lack of detailed search strategies, inclusion and exclusion criteria, and formulation methods in the guidelines are the reasons for the differences. Additionally, some guidelines [4, 6, 7] have low reliability because of a lack of support for the relevant evidence, which may be because the guidelines were published earlier, as there was no complete standard for guidelines at that time.

Almost all nine guidelines scored highly in the domain of clarity and presentation, with clear and concise recommendations, highlighted marks, and a number of charts and tables, which are easy to understand and apply, showing reasonable and commendable development of the guidelines for malignant pleural effusion. However, some guidelines [2, 3, 9] still contain ambiguous recommendations due to insufficient evidence and need to be expanded by searching for more high-quality evidence and research in the future.

The lack of consideration of promotions and obstacles in application and potential resource costs led to low scores in the domain of applicability in nine guidelines. In fact, it is worth taking applicability into consideration, and the pros and cons should be carefully analyzed when making guidelines, such as language limitations, regional epidemiological differences, whether the local medical conditions are suitable for conducting these operations, the urgent need for recommendations in clinical treatment, and the value of increasing budget and funds. Then, we assessed the applicability of the guidelines, ensuring that they can be used for most populations.

Editorial independence is a domain that aims to verify the equality and objectivity of the guidelines; unfortunately, most of the guidelines do not mention whether external sponsorship was available. This is not beneficial for the participants, as the guidelines could be suspected to have commercial interests.

In general, each guideline has room for improvement in different domains, especially in domain 2 and domain 5, which were usually ignored by most of the guidelines. Second, the quality of evidence needs more attention, and high-quality evidence and grading systems are key to helping clinical workers find the most suitable treatment for patients with MPE or malignant ascites.

### Heterogeneity and reasons for these differences in the recommendations among guidelines for the management of malignant pleural effusions

The description and presentation of recommendations are the focus of rigor of development, but the rigor of development fail to evaluate the content of recommendations. The recommendations are the core content of guidelines. Users of guidelines pay more attention to the specific content of the recommendation. The specific content and strength of recommendation are closely related to the research content and quality of supporting evidence. Therefore, it is necessary to compare the consistency of the key recommendations in the guidelines and find out the reasons for the heterogeneity of recommendations from the perspective of supporting evidence.

The findings of heterogeneity and reasons for these differences in the recommendations among guidelines in this study are different to the findings in a previous study [14]. Although the previous study focused on the heterogeneity of nutritional care procedures [14], which was obviously different from this study, the results of the two studies were similar in terms of heterogeneity and reasons for main recommendations.

#### Different recommendation content and emphasis for MPE guidelines

After sorting out the main recommendations of eight guidelines, we found that IPC (indwelling pleural catheter) and chemical pleurodesis were recommended by almost all the guidelines, and the most effective sclerosant for chemical pleurodesis is talc. In addition, treatment for special conditions and other complications were recommended in some guidelines but rarely mentioned in other guidelines. For example, the reference guidelines [5] makes a recommendation for the management of IPC-associated infection, considering antibiotic treatment first and removing the catheter after failing to control the infection. Another set of guidelines [2] concluded that intrapleural fibrinolytic agents for septated and loculated MPE cannot change the clinical outcomes even though they improve the fluid drainage volume. Moreover, these guidelines also recommend factors that predict can prognosis for patients with malignant pleural effusion and recommend a diagnosis for MPE. In their view, histological diagnosis has low sensitivity, and pleural biopsy is still recommended as the gold standard. Additionally, oncology treatment is discussed in the guidelines; unfortunately, there is a lack of sufficient evidence to support oncology treatment, and the guidelines still do not provide a definite answer to this question. Another set of guidelines [3] provided the largest number of recommendations, including the management of analgesia, premedication, malignant seeding, clamp and removal of the intercostal tube, intrapleural fibrinolytics, thoracoscopy, and long-term IPC drainage, and recommended that rotation is unnecessary after chemical pleurodesis. Some guidelines [1] recommend rmhTNF treatment alone or in combination with some anti-tumor drugs, which has been supported by much clinical evidence. Other guidelines [7] recommend parenteral fluid management in terminal MPE patients but lack evidence. Another set of guidelines [8] do not recommend the use of diuretics for MPE patients.

#### Controversy in the recommendations for the management of malignant pleural effusion

##### Should thoracentesis be performed in patients with MPE?

In patients with symptomatic MPE, most guidelines [3–6, 8] recommend thoracentesis under the guidance of ultrasound to improve the symptoms. The highest evidence for this recommendation is from a prospective observational cohort study [16] graded as B/2b in OCEBM. Additionally, different guidelines for this issue have different emphases. For example, in the reference guidelines [5], ultrasound-guided thoracentesis is recommended in patients with a definite diagnosis or suspicion of malignant pleural effusion (MPE), while two other guidelines [3, 4] clearly suggest that thoracentesis is not recommended in patients with a life expectancy of more than 1 month due to the high recurrence rate of pleural effusion. However, these two guidelines [3, 4] do not provide corresponding evidence for this recommendation, making it impossible for us to judge the evidence sources of the two recommendations.

##### Should talc poudrage or talc slurry be used for chemical pleurodesis in patients with malignant pleural effusion?

Six guidelines [2–6, 8] clearly and consistently recommend chemical pleurodesis as a treatment for malignant pleural effusion. Five of these guidelines [2–6] provide recommendations on the use of talc poudrage or talc slurry in chemical pleurodesis, but we found some disagreements. Three guidelines [3–5] think talc poudrage and talc slurry have the same effect. In another set of guidelines [2], talc poudrage is considered better. However, the supporting evidence for these recommendations in these guidelines is similar and mainly originates from four systematic reviews [17–20]. One of the studies [19] thought that talc poudrage can reduce the recurrence of pleural effusion compared with talc slurry, but only 2 studies were included, and the sample size was obviously insufficient. According to network meta-analysis results, talc poudrage was better than slurry in controlling pleural effusion. In another systematic review [18], the authors included three studies in the subgroup analysis and found no difference between talc poudrage and talc slurry in the success rate of pleurodesis, but talc poudrage was associated with more respiratory complications (*P* = 0.003) (heterogeneity test: *I*^2^ = 4.79%). In the last systematic review [17], the author included four studies into a subgroup analysis and found that talc poudrage has a higher success rate in pleurodesis than talc slurry (*P* = 0.026) (heterogeneity test: *I*^2^ = 16.5%, *P* = 0.309). Based on the above evidence, talc poudrage has a slight advantage over talc slurry; although the recommendations differ slightly in their wording, we tend to think the two statements are consistent. Furthermore, there are still unreasonable citations of evidence in the guidelines. The main evidence cited in one set of guidelines [3] are RCT studies, one of which has only an abstract [21], and the other was a large sample RCT study [22] (graded A/1b in OCEBM); thus, these guidelines lacked the results of systematic review. Other guidelines [4] do not provide any specific evidence. A set of guidelines [6] recommended talc slurry only but did not mention talc poudrage, and no evidence was provided.

##### Should large-bore tubes or small-bore tubes be used in pleurodesis or fluid drainage?

Three guidelines [2–4] recommend choosing bore tubes, but the recommendations differ significantly. Some guidelines [3] recommend the use of small-bore tubes (10–14 F), and the supporting evidence is mainly from two low-quality RCTs (graded B/2b in OCEBM). One of which is an RCT of 41 people [23], suggesting almost no difference in the success rate of pleurodesis between small- and large-bore tubes. The other RCT [24], involving 18 cases, also suggested that the efficacy of small- and large-bore tubes was the same, but there was less discomfort with small-bore tubes. Instead, other guidelines [2] recommend large-bore tubes (e.g., 24 F), and the main evidence to support its conclusion is a large sample of RCTs [25] that included 320 patients. The study found that large-bore tubes (24 F) had a higher success rate of pleurodesis than small-bore tubes (12 F). However, the postoperative pain scores in patients with small-bore tubes (12 F) were lower than that in patients with large-bore tubes (24 F).

However, one set of guidelines [4] believes that the effect of large-bore tubes and small-bore tubes is equivalent, and there is no significant difference in the success rate of fluid drainage and sclerosant injections; however, the discomfort of small-bore tubes is mild, so small-bore tubes were recommended for use under the guidance of ultrasound, but no relevant evidence was provided.

Regarding the level of evidence, there is still a lack of sufficient high-quality RCTs to confirm the advantages and disadvantages of large- and small-bore tubes in pleurodesis.

#### Problems with the recommendations and supporting evidence for the management of malignant ascites

The guidelines for malignant ascites are quite rare at present, and we only identified three relevant guidelines [1, 7, 9]. Moreover, it is difficult for us to summarize the recommendations and evidence because the three guidelines have completely different emphases on the management of malignant ascites. In general, one set of guidelines [1] recommended the use of rmhTNF alone and some combination of anti-tumor agents for the treatment of malignant ascites, and the strength of the recommendation and the level of evidence were graded as B and2b, respectively, in OCEBM. Another set of guidelines [7] also recommended parenteral fluid management in patients with malignant ascites, but no evidence was provided to support this. The third set of guideline [9] suggested the use of diuretics such as spironolactone and furosemide, abdominal paracentesis, and indwelling peritoneal catheters, but there is insufficient evidence to support this recommendation. Whether cell-free and concentrated ascites reinfusion therapy can be applied remains unclear. The other treatment of Denver peritoneovenous shunts is not recommended due to its association with multiple and serious complications. The recommendation strength and evidence level of the above suggestions according to OCEBM are both C/4. In summary, there is much controversy regarding the clinical management of malignant ascites, which urgently needs more research and guidelines.

### Suggestions for the formulation of guidelines

In view of the above problems, this study puts forward some suggestions for future guidelines: (1) in the process of formulating guidelines for the treatment of malignant pleural effusion, a systematic search strategy for evidence is recommended, and explicit items of recommendation should be set as comprehensively as possible. (2) Additionally, to help readers and users to locate the evidence, reliable evidence sources are required. (3) Controversial recommendations, for example, whether to recommend talc poudrage or talc slurry for patients with MPE and which treatment is better, should depend on the quality of evidence. A conservative recommendation tends to be suggested when the quality and quantity of evidence cited is very low. (4) There is a lack of guidelines for the management of malignant ascites at present; more evidence-based guidelines should be created to guide the management of malignant ascites in the future. (5) In the process of formulating guidelines, a uniform reference standard is required to reduce the heterogeneity among guidelines, and the AGREE , tool is worth recommending. (6) In addition, guidelines should also include the suggestions and feelings of the target patients and express concern and appreciation for these patients. (7) Preferably, future guidelines should reach an agreement for international cooperation, discuss the heterogeneity among guidelines written by different organizations and countries, and give a complete and universal version of the guidelines for MPE and malignant ascites.

### Advantages and limitations of the study

There are some advantages in our study: (1) we used the AGREE IIGREE dto objectively assess MPE and malignant ascites treatment guidelines, providing a great reference for the future formulation of guidelines, and (2) to analyze the heterogeneity among recommendations, the OCEBM grading system was used to reclassify relevant evidence, and we also found the reasons for such heterogeneity in the current guidelines. This study has some limitations: (1) we included only guidelines published in English and Chinese and did not provide a comprehensive evaluation of the guidelines published in other languages. (2) The guidelines we considered were from the last 10 years. Within 10 years, the updated evidence and treatment have contributed to the heterogeneity of the recommendations. (3) The guideline quality evaluation based on the AGREE based mainly focused on the methodology but lacked a content and evidence assessment; this led to low-quality guidelines scoring very high and being used as a reference. Even though the total score cannot fully reflect the true quality of the guidelines, we used the OCEBM grading system to analyze the strength of the recommendations and level of evidence; to some extent, this grading system remedied the defects of the AGREE s tool. (4) In this study, only the guidelines for the management of malignant pleural effusion and ascites were included, and the results can only show the problems and quality of the current guidelines for the diagnosis and treatment of malignant pleural effusion and ascites, but fail to represent the current quality and problems of the guidelines related to thoracic surgery.

## Conclusion

This quality assessment of guidelines for malignant pleural effusion and malignant ascites revealed the differences and problems among different guidelines. The quality of the guidelines was affected by the rigor of development, in which the level of evidence varied greatly among guidelines, and there was also a lack of an appropriate evidence grading system in some guidelines. Furthermore, the two domains of stakeholder involvement and applicability are insufficient and disappointing in most of the guidelines. Moreover, regarding the content of the recommendations, there is some argument and controversy regarding the choice between talc poudrage and talc slurry and between large-bore tubes and small-bore tubes. In addition, each set of guidelines includes some unique treatment in the management of malignant ascites. These heterogeneities may be due to the different preferences of the guideline makers, the poor quality of evidence, and the limited level of medical treatment at present. Hopefully, these issues will be better addressed in future guidelines and updates.

## Data Availability

All data generated or analyzed during this study are included in this published article.
